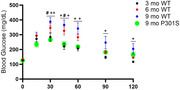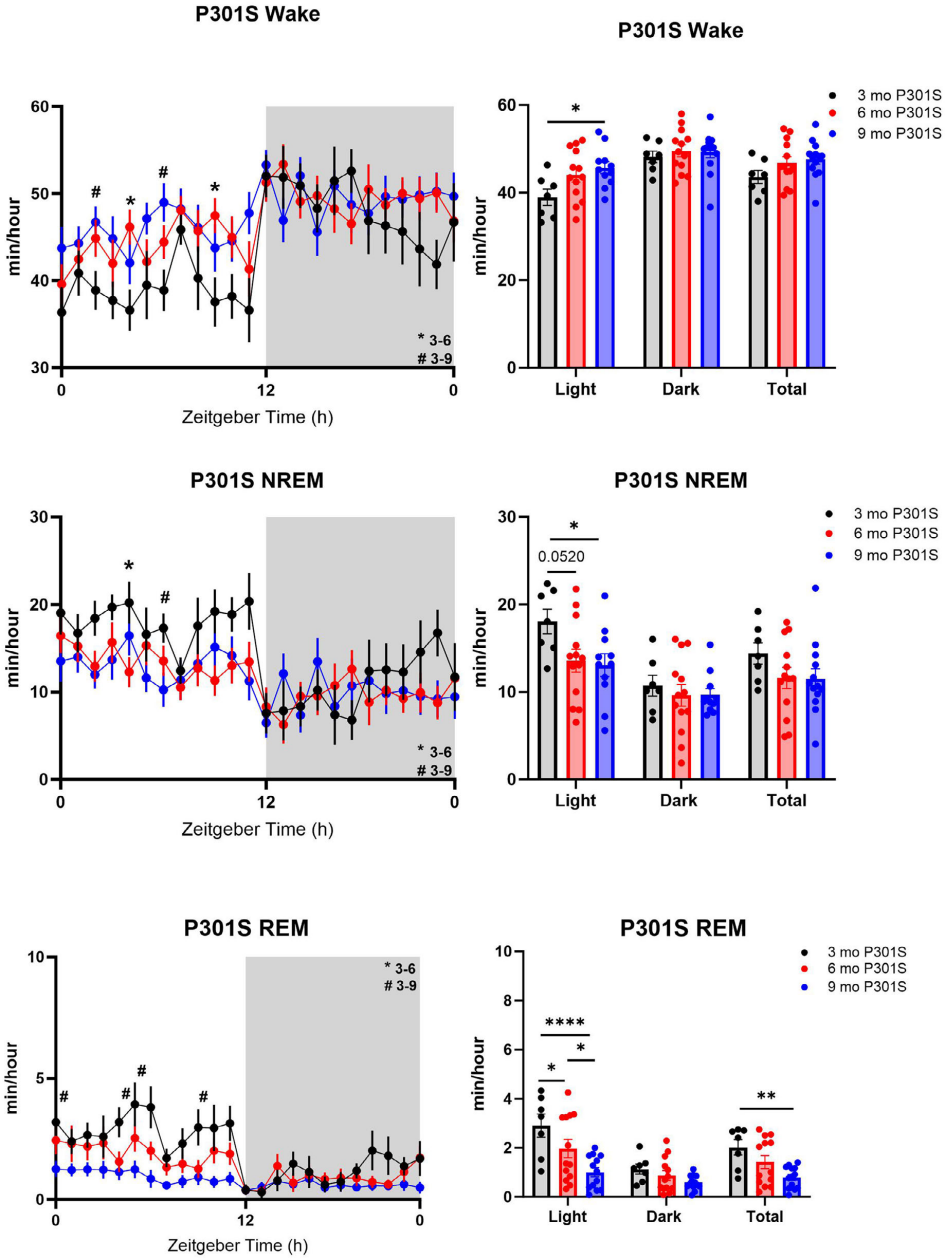# Tau pathology induced alteration in neuronal activity disrupt sleep and cerebral metabolic rhythms in the P301S PS19 mouse model of tauopathy

**DOI:** 10.1002/alz.086613

**Published:** 2025-01-03

**Authors:** Riley E Irmen, Caitlin M Carroll, Nicholas J Constnantino, James A Snipes, Shannon L Macauley

**Affiliations:** ^1^ University of Kentucky, Lexington, KY USA; ^2^ Wake Forest University School of Medicine, Winston Salem, NC USA

## Abstract

**Background:**

Alzheimer’s disease is defined by the pathological aggregation of amyloid‐beta and hyperphosphorylated tau. AD patients often exhibit other symptoms like metabolic and sleep dysfunction. Currently, it is unclear if impairments are a cause or consequence of Aβ or tau aggregation. Previously, we demonstrated cerebral and peripheral changes in glucose elevate brain lactate levels stimulating wake. Further, previous work showed tau pathology corresponds with decreased sleep. Currently, it’s unclear what mechanisms drive sleep changes or if it relates to metabolic dysfunction. Therefore, we investigated how tau pathology impacts sleep and metabolism in P301S mice.

**Methods:**

To examine peripheral metabolism, glucose tolerance tests were conducted on P301S and wild‐type mice(female). Recordings of indirect calorimetry over 3 days further explored peripheral metabolism. Paired glucose and lactate biosensors in the hippocampi tracked sub‐second fluctuations of brain interstitial fluid (ISF) to observe cerebral metabolic changes. EEG and EMG recorded cortical activity over a 3‐day period and spectral analysis investigated sleep/wake architecture.

**Result:**

Tau aggregation preserves peripheral glucose tolerance and diurnal rhythms of brain glucose/lactate, normally lost in aged controls, suggesting increased glucose utilization. Similarly, P301S mice show increased respiratory exchange ratio suggesting heightened carbohydrate utilization. Conversely, they exhibit diminished energy expenditure. EEG spectral analysis revealed increased delta, increased theta, and decreased beta with pathology, suggesting disrupted GABA activity and sleep drive. Starting early in pathology, mice display increased wake and decreased NREM/REM. Effects on total sleep depend heavily on decreased REM bout number and duration.

**Conclusion:**

Blood glucose levels in P301S mice fail to rise in response to glucose injection. Brain glucose/lactate fluctuations are maintained suggesting tau pathology preserves glucose utilization. Conversely, decreased NREM and REM and increased wake were exhibited with tau pathology. EEG spectral analysis indicates decreased beta activity, suggesting diminished GABA activity. Increased theta during NREM suggests increased pressure to transition to REM sleep. Reduced sleep drive is illustrated by increased delta in wake. Together, tau pathology reduces sleep drive and ability to switch between vigilant states despite increased sleep need. These results suggest that tau pathology causes excitatory/inhibitory imbalance and sleep impairment but does not contribute to profound effects on cerebral metabolic rhythms.